# Cell Type-Dependent Induction of DNA Damage by 1800 MHz Radiofrequency Electromagnetic Fields Does Not Result in Significant Cellular Dysfunctions

**DOI:** 10.1371/journal.pone.0054906

**Published:** 2013-01-23

**Authors:** Shanshan Xu, Guangdi Chen, Chunjing Chen, Chuan Sun, Danying Zhang, Manuel Murbach, Niels Kuster, Qunli Zeng, Zhengping Xu

**Affiliations:** 1 Bioelectromagnetics Laboratory, Zhejiang University School of Medicine, Hangzhou, China; 2 Institute of Occupational Health Assessment, Guangdong Prevention and Treatment Center for Occupational Disease, Guangzhou, China; 3 Foundation for Research on Information Technologies in Society, Swiss Federal Institute of Technology, Zurich, Switzerland; National Institute on Aging, United States of America

## Abstract

**Background:**

Although IARC clarifies radiofrequency electromagnetic fields (RF-EMF) as possible human carcinogen, the debate on its health impact continues due to the inconsistent results. Genotoxic effect has been considered as a golden standard to determine if an environmental factor is a carcinogen, but the currently available data for RF-EMF remain controversial. As an environmental stimulus, the effect of RF-EMF on cellular DNA may be subtle. Therefore, more sensitive method and systematic research strategy are warranted to evaluate its genotoxicity.

**Objectives:**

To determine whether RF-EMF does induce DNA damage and if the effect is cell-type dependent by adopting a more sensitive method γH2AX foci formation; and to investigate the biological consequences if RF-EMF does increase γH2AX foci formation.

**Methods:**

Six different types of cells were intermittently exposed to GSM 1800 MHz RF-EMF at a specific absorption rate of 3.0 W/kg for 1 h or 24 h, then subjected to immunostaining with anti-γH2AX antibody. The biological consequences in γH2AX-elevated cell type were further explored with comet and TUNEL assays, flow cytometry, and cell growth assay.

**Results:**

Exposure to RF-EMF for 24 h significantly induced γH2AX foci formation in Chinese hamster lung cells and Human skin fibroblasts (HSFs), but not the other cells. However, RF-EMF-elevated γH2AX foci formation in HSF cells did not result in detectable DNA fragmentation, sustainable cell cycle arrest, cell proliferation or viability change. RF-EMF exposure slightly but not significantly increased the cellular ROS level.

**Conclusions:**

RF-EMF induces DNA damage in a cell type-dependent manner, but the elevated γH2AX foci formation in HSF cells does not result in significant cellular dysfunctions.

## Introduction

With the rapid development of mobile communications, e.g., globally estimated 5.9 billion mobile phone subscriptions at the end of 2011 [1], exposure to radio frequency electromagnetic fields (RF-EMF) emitted by mobile telephony has become a major public health concern. Therefore, it is necessary to investigate and understand its potential health impact.

Most epidemiological investigations have looked for an association between brain tumors and mobile phone use, and the overall evidence indicates an increased risk despite several methodologic concerns [2]. Recently, the INTERPHONE report on gliomas and meningiomas was released [3]. Although the data suggest an increased risk of glioma at the highest exposure levels, the authors conclude that biases and errors prevent a causal interpretation. The International Agency for Research on Cancer (IARC) working group then classifies RF-EMF as “possibly carcinogenic to humans” (Group 2B), in view of the limited evidence in humans and in experimental animals [4]. It seems that more reliable epidemiological methodology is required to elucidate the impact of this environmental factor on human health. Moreover, it would be helpful to interpret the reported epidemiological findings if a genotoxicity of RF-EMF could be firmly established. For this reason, numerous *in vitro* and *in vivo* studies have examined its genotoxic potential. Although a number of studies report a DNA damage effect of RF-EMF, the overall results remain contradictory and inconclusive [5]–[7].

Two major issues might account for the current controversies. First, different research groups have used different cell types, exposure systems and exposure parameters that make it difficult, if not impossible, to compare the data. Second, the key technique used in the majority of the genotoxic studies was the comet assay, which detects the late consequences of DNA damage. Despite having several advantages over other technologies, the comet assay also has a number of limitations that may hamper the interpretation of the results [8], [9]. Meanwhile, there are technical challenges in replicating this method exactly, which may also lead to discrepancies in the results [5]. Therefore, analysis of the effect of RF-EMF on DNA damage by more sensitive and controllable methods to reveal any subtle impact, and to carry out the experiments in a systematic way to make the data comparable are urgently needed. As a matter of fact, further studies on the influence of genetic background and cell type using newer, more sensitive methods has been identified as one of the research needs in the 2010 World Health Organization Research Agenda for Radiofrequency Fields.

Recently, the method of immunofluorescent visualization of γH2AX (the phosphorylated form of histone H2AX) foci formation has become a sensitive and specific method to detect DNA double-strand breaks (DSBs) [10], [11]. Phosphorylation of H2AX is one of the earliest marks of DSBs in eukaryotes [12]. It has been shown that after exposure to ionizing radiation or other DSB-inducing factors, thousands of H2AX molecules become rapidly phosphorylated to γH2AX at the site of each nascent DSB. The γH2AX can be visualized as discrete foci by immunofluorescent staining with a specific antibody against γH2AX and counted under a fluorescent microscope. It has been demonstrated that each γH2AX focus indicates a single DSB [13], [14], and the γH2AX assay is capable of detecting DNA damage at a sensitivity 100-fold higher than that of the comet assay [15]. This potential to visualize single focus within a nucleus makes γH2AX immunofluorescent staining a sensitive available method for detecting DSB in cells [14], [16]. Since its first introduction to measure the effect of RF-EMF from the Global System for Mobile Communication (GSM) mobile phone on chromatin conformation [17], the γH2AX assay has proven to be a feasible technique in detecting the effect of RF-EMF on DNA damage in three different cell types [18]–[20].

To systematically investigate the effects of RF-EMF on DNA damage, we examined and compared γH2AX foci formation in six cell types after GSM 1800 MHz RF-EMF exposure. Since the γH2AX foci formation is an early marker of DNA damage, we next determined whether the exposure-induced γH2AX foci formation resulted in genetic instabilities, aberrant cell cycle progression and other cellular dysfunctions.

## Materials and Methods

### Ethics Statement

This study was conducted according to the principles expressed in the Declaration of Helsinki. The use of human umbilical cord was approved by the Ethics Committee of Zhejiang University School of Medicine (Approval ID 2010-121). All mothers provided written informed consent for the collection of their umbilical cords and subsequent analysis.

All procedures for the isolation of rat astrocytes were reviewed and approved by the Animal Ethics Committee at Zhejiang University (Approval ID ZJU2009102020). Efforts were made to reduce animal suffering and the number of animals used.

### Exposure System

The exposure system has been described previously [21]. It is based on a dual resonant R18-waveguide setup for six 35 mm Petri dishes positioned in the H-field maximum of the standing wave that has been developed and provided by the Foundation for Information Technologies in Society (IT’IS Foundation, Zurich, Switzerland) together with the calibration, dosimetric and temperature data. One of the waveguide is excited by an RF-EMF signal mimicking the basic pulse structure of the GSM signal at 1800 MHz, i.e., pulsed amplitude modulation with a pulse width of 0.576 ms and a repetition rate of 217 Hz. Details of the waveguide system and the dosimetry including the distribution of specific absorption rate (SAR) and temperature are documented in the literature [22]. In the other waveguide the Petri dishes are placed for sham exposure (isolation to exposure waveguide >60 dB). Due to the different dielectric properties of the medium (relative permittivity = 71 and conductivity = 1.75 (1.68–1.84) S/m) used in this study than the Dulbecco’s Modified Eagle Medium (DMEM) evaluated in the original dosimetric assessment [22], SAR and temperature distribution were reevaluated. We applied the full 3-D electromagnetic and thermal simulation platform SEMCAD X V14.8 (SPEAG, Zurich, Switzerland) to the detailed exposure system model shown in Figure S1. The bottom 50 µm of the medium was evaluated to determine the macroscopic exposure of the cell monolayer. The minimal voxel dimensions were 50 µm, whereas the voxel size did not exceed 300 µm in the medium, 700 µm in the whole petri dish, and 4 mm in the entire simulation space. All material parameters applied in the original dosimetric assessment [22] except for the dielectric parameters were also applied in the reevaluation. The average exposure of SAR/H^2^ was 0.64 W/kg/(A^2^/m^2^), confirming the extrapolation from the original one [22]. The uncertainty of the dosimetric assessment was estimated to 20% (k = 1) and the variability of less than 30%, the details of the assessment is provided in [22]. The air temperature inside the waveguides was measured by the sensor at the air exceed of the waveguides which remained at 37±0.1°C during the whole duration of cell exposure. The temperature distribution within the Petri dishes was verified using temperature probes and temperature analysis in terms of the incident field strength, cell medium volume, and air flow [22]. The temperature rise was assessed by measurement and simulation, and a maximum rise of <0.03°C/(W/kg) for monolayer cells was documented [22]. Thus, the temperature rise was less than 0.1°C under the exposure of SAR at 3.0 W/kg.

### Cell Cultures

Chinese hamster lung cells (CHLs) were from the cell bank of the Shanghai Institutes for Biological Sciences, Shanghai, China, and grown in RPMI 1640 medium (Invitrogen, Carlsbad, CA, USA) supplemented with 10% heat-inactivated fetal bovine serum (FBS, Si Ji Qing, Zhejiang Tianhang Biomedical Technology Co., Ltd., Hangzhou, China).

Primary newborn Sprague-Dawley rat astrocytes were isolated from the cerebral cortex of newborn rats (1-day-old) as described by Suder et al. [23] with minor modification. Briefly, 1-day-old rats were decapitated and their brains were immersed in phosphate-buffered saline (PBS) on ice. After carefully removing the meninges, the cortex was cut into small pieces, and trypsin with ethylene diamine tetraacetic acid (EDTA) was added to a final concentration of 0.25%. After incubating at 37°C for 20 min, DMEM and Ham’s F-12 Nutrient Mixture (1∶1) (Hyclone, Thermo Scientific, Beijing, China) supplemented with 20% FBS (Hyclone, Thermo Scientific), 100 units/mL penicillin (Invitrogen), and 100 µg/mL streptomycin (Invitrogen) were added to stop the process. Five minutes later, the tissue pellet was transferred into fresh medium and resuspended with a fire-polished pipette. After centrifugation, the supernatant was mixed with fresh culture medium and plated at 1×10^7^ cells per 100-mm-diameter Petri dish coated with poly-D-lysine (PDL, Sigma, St. Louis, MO, USA). After a 15 min differential attachment, suspended cells were transferred to new PDL-coated dishes. Cells were maintained at 37°C in a humidified atmosphere of 5% CO_2_ for 7–9 days until a confluent monolayer was formed, then the dishes were shaken at 250 rotation per minute (rpm) on a rotary shaker at 37°C for at least 18 h to remove micro- and oligo-dendroglia from the astrocytes. The astrocytes were characterized at 14 days by immunoﬂuorescent staining for glial fibrillary acidic protein (GFAP) with anti-GFAP antibody (Zhongshan Golden Bridge Biotechnology Co., Ltd., Beijing, China). The purity of isolated astrocytes was >96%.

Human amniotic epithelial cells (FLs) were from American Type Culture Collection (ATCC, Manassas, VA, USA), and grown in Minimum Essential Medium (MEM, Invitrogen) supplemented with 10% heat-inactivated FBS (Zhejiang Tianhang Biomedical Technology Co., Ltd.).

Human lens epithelial cells (HLECs) SRA01/04 were from the Riken Cell Bank, Tsukuba, Ibaraki, Japan, and grown in low glucose DMEM (Invitrogen) supplemented with 20% heat-inactivated FBS (Zhejiang Tianhang Biomedical Technology Co., Ltd.).

Human skin fibroblasts (HSFs) were from the Lawrence Berkeley National Laboratory, Berkeley, California, USA, and grown in α-MEM (Invitrogen) supplemented with 10% heat-inactivated FBS (Zhejiang Tianhang Biomedical Technology Co., Ltd.).

Human umbilical vein endothelial cells (HUVECs) were isolated from human umbilical cords as reported by Marin et al. [24] with minor modification. With the mother’s informed consent, the freshly-obtained umbilical cord from a normal placenta was rapidly immersed in PBS and processed for endothelial cell isolation within 12 h. Under a sterile laminar-flow hood, the umbilical vein was cannulated and thoroughly rinsed with PBS, and then trypsin (0.25%) was injected at one end of the vein, while the distal end was tightly clamped with surgical clips. During 10 min incubation at 37°C, the cord was gently squeezed for several times. The trypsin solution was then discarded, and the vein was gently washed with 30 mL PBS that was collected in a 50 mL sterile polypropylene tube. The collected cells were pelleted by centrifugation for 5 min at 2000 rpm, and the cell pellet was gently resuspended in Human Endothelial-serum free medium (SFM, Invitrogen) containing 10% FBS (Invitrogen), 15 µg/mL endothelial cell growth factor (Sigma), 100 units/mL penicillin (Invitrogen), and 100 µg/mL streptomycin (Invitrogen). Half of the culture medium was-changed after 6 h and confluence was typically achieved in 6–8 days with a “cobblestone” appearance under optical microscopy. The cells were characterized by immunoﬂuorescent staining for von Willebrand factor (VWF) with anti-VWF antibody (Gene Tech Co., Ltd., Shanghai, China) and platelet endothelial cell adhesion molecule (PECAM) with anti-PECAM antibody (Abcam, Cambridge, UK). The purity of isolated HUVECs was >98%. The cells were then grown in 52.5% M199 medium (Thermo Scientific) and 36% Human Endothelial-SFM (Invitrogen) supplemented with 10% FBS (Invitrogen), 15 µg/mL endothelial cell growth supplement (Sigma), 100 units/mL penicillin (Invitrogen), and 100 µg/mL streptomycin (Invitrogen). The cells in passages 2–8 were used in the experiments.

### Cell Exposure Protocol

All cell types used in the study were cultured at 37°C in a humidified atmosphere of 5% CO_2_ to ∼80% confluence, and then sub-cultured by plating 1×10^5^ cells (except for CHL which was plated at 5×10^4^) per 35-mm-diameter Petri dish (NUNC, Thermo Scientific, Roskilde, Denmark) containing a glass coverslip on the bottom. Twenty-four hours after the seeding, cells were divided into sham-exposure, 1 h exposure, and 24 h exposure groups. The sham group was sham exposed for 24 h. The 1 h exposed group was first sham exposed for 23 h and then exposed to RF-EMF for 1 h. The 24 h exposure group was exposed to RF-EMF for 24 h. Using this setup it was possible to prepare all cell cultures for the exposures at the same time and from the same batches of the respective cell types. This approach allowed us to avoid the possible variability of cell cultures that might be caused by differences between batches, differences that might be caused by the confluence level of cultures and differences in the cell cycle distribution of cells within the culture. This setup also allowed the use of the same sham sample as a control for both the 1 h and the 24 h exposures.

The cultures were exposed to 1800 MHz RF-EMF at an average SAR of 3.0 W/kg for 1 or 24 h using an intermittent exposure cycle consisting of periods of 5 min of radiation “on” followed by 10 min of radiation “off”. This intermittent scheme, simulating the intermittency of real exposures, was first introduced in the ELF study which showed it had stronger effect than continuous exposure [25], and then replicated and extended by Focke et al [26]. It has been applied to RF exposures [21], [27]–[29]. As a positive control, we treated cells for 1 h with 1 µM 4-nitroquinoline 1-oxide (4NQO, Sigma) to induce DNA DSBs. This experiment was done in a blind manner by one group of experimenters who carried out the exposures and another group who did the assays independently.

To expose the cells at an average SAR of 3.0 W/kg, we first measured the dielectric parameters of each type of complete medium and determined the incident fields necessary to obtain the target SAR values. The actual measured field values were used for feed-back regulation of the output power of the RF generator to achieve the desired SAR.

### Immunofluorescence Staining

Immunofluorescent detection of γH2AX was performed as described previously [30] with minor modifications. Immediately after the exposure, cells were fixed in 4% paraformaldehyde for 15 min at 4°C, and permeabilized in 0.5% Triton X-100 for 15 min at 4°C. Non-specific binding sites were blocked with blocking serum (Zhongshan Golden Bridge Biotechnology Co., Ltd.) for 2 h. The cells were then incubated with a mouse monoclonal anti-γH2AX antibody (Upstate, Millipore, Temecula, CA, USA; diluted 1∶1000) for 2 h, and incubated with tetramethyl rhodamine isothiocyanate-conjugated goat-anti-mouse secondary antibody (Zhongshan Golden Bridge Biotechnology Co., Ltd.; diluted 1∶300) for 1 h. Thereafter, the samples were incubated with 4′, 6-diamidino-2-phenylindole (DAPI, Sigma) and stained for 15 min to visualize the nuclei. Each step was followed by 3 washes for 5 min in PBS. Then, the cover slip was removed from the Petri dish and mounted onto a glass slide.

Images were recorded from 5 to 10 fields that were randomly selected from two slides under an Olympus AX70 fluorescent microscope (Olympus, Tokyo, Japan) with a 40× oil immersion objective. About 200 cells were analyzed for each independent exposure experiment and for each exposure condition (type of cell line, duration of exposure), and the number of foci per cell was used as the index of DSBs. Each experiment was independently repeated at least four times.

### Alkaline and Neutral Comet Assay

The comet assay is a microgel electrophoresis technique that measures the fragmented chromosomal DNA, an index of genome instability, as a late consequence of DNA damage at the level of single cells. There are two different comet assays depending on the pH of the electrophoresis performed: alkaline pH for a broad spectrum of DNA strand breaks, and neutral pH for DSBs [31], [32]. We performed both alkaline and neutral comet assays in HSF cells as described by Lai and Singh [33], [34] with minor modification. Cells detached with 0.25% trypsin-EDTA were resuspended in ice-cold PBS, mixed with pre-warmed (37°C) 0.65% low-melting agarose, rapidly loaded onto slides pre-coated with 0.65% normal-melting agarose and cooled in ice. Slides were immersed in an ice-cold lysis solution (2.5 M NaCl, 1% sodium N-lauroyl sarcosinate, 100 mM disodium EDTA, 10 mM Tris base, pH 10) containing 1% Triton X-100 for 1 h, then treated with DNase-free proteinase K (0.5 mg/mL, Amresco, OH, USA) in lysis solution without Triton X-100 for 2 h at 37°C. To detect DSBs by the neutral comet assay, after lysis, slides were treated with ribonuclease A (RNase A, 10 µg/mL, Amresco) and then with proteinase K (0.5 mg/mL) both in the lysis solution without Triton X-100 for 2 h at 37°C. Subsequently, the slides were subjected to electrophoresis at 300 mA (20 V, 0.4 V/cm) for 20 min after 20 min unwinding. The electrophoresis buffers (alkaline comet assay: 300 mM NaOH, 0.1% 8-hydroxyquinoline, 2% dimethylsulfoxide, 10 mM tetrasodium EDTA, pH 13; neutral comet assay: 100 mM Tris, 300 mM sodium acetate, and acetic acid at pH 9.0 ) were ice-cold and the electrophoresis apparatus was sitting on ice. After electrophoresis, the slides were neutralized with Tris buffer (0.4 M, pH 7.5), and air-dried. Comets were stained with Gel-Red (Biotium, CA, USA), and examined under an Olympus AX70 fluorescent microscope with a 20× objective. All procedures were performed in the dark. About 200 cells were analyzed for each independent exposure experiment, and DNA damage parameters were calculated using CASP 1.2.2 software (Krzysztof Konca, Wroclaw, Poland) [35], [36]. The tail DNA was calculated as the percentage of DNA in the comet tail, the tail length was calculated as the length of the comet tail measured from the right border of the head area to the end of the tail (in pixels) and the tail moment was calculated as tail DNA × tail length. As a positive control, we treated cells for 1 h with 0.02 µM 4NQO to induce DNA damage. Each experiment was independently repeated for eight (neutral) or twelve (alkaline) times and two dishes were included per exposure condition.

### TUNEL Assay

Since the γH2AX foci formation assay is an indirect method to evaluate DNA damage, we also performed the TdT-mediated dUTP Nick-End Labeling (TUNEL) assay to examine the presence of DNA nicks in HSF cells using DeadEnd Fluorometric TUNEL System (Promega, Madison, USA). Briefly, the cells were washed twice with PBS and fixed with 1% methanol-free formaldehyde solution in ice-cold PBS for 20 min and permeabilized with 70% of ethanol in PBS at −20°C over night. Before TUNEL reaction labeling, the cells were incubated in equilibration buffer at room temperature for 5 min. The TUNEL reaction labeling was performed according to the protocol provided by the manufacture and the cells were incubated in a dark humidified chamber at 37°C for 60 min. To stop the reaction, the cells were incubated in the 20 mM EDTA buffer. The cells were then collected by centrifugation, and were resuspended in 0.1% Trition X-100 solution in PBS containing 5 mg/mL BSA. The fluorescein-12-dUTP-labeled DNA was quantitated by flow cytometry (Beckman Coulter, CA, USA), in which 10,000 events per sample were acquired. As a positive control, we treated cells with 1 µM 4NQO for 1 h to induce DNA fragment formation. Each experiment was repeated for three times and six dishes were included per exposure condition.

### Cell Cycle Analysis

After culturing for an additional 0, 6 or 12 h after 24 h RF-EMF exposure, cells were detached with 0.25% trypsin-EDTA and then resuspended in 70% ethanol at −20°C. Fixed cells from independent experiments were stored together at −20°C before measurement. Cells were stained with 50 µM propidium iodide (sigma) in 500 µl PBS buffer containing 10 µg/mL RNase A and 0.2% Triton X-100 for 30 min at room temperature in the dark. The cell cycle progression was assessed by flow cytometry, in which 10,000 events per sample were acquired, and the percentage of cells in the G0/G1, S and G2/M phases were determined using Wincycle32 software (Beckman Coulter). Each experiment was repeated five times and two dishes were included per exposure condition.

### Cell Proliferation Assay

The numbers of cells detached with 0.25% trypsin-EDTA were measured by a Z1 Coulter Particle Counter (Beckman Coulter) at an additional 0, 12, 24 or 48-h culture after 24 h RF-EMF exposure. As a positive control, we treated cells with 1 µM 4NQO for 1 h to inhibit cell proliferation. Each experiment was repeated three times and two dishes were included per exposure condition.

### Cell Viability Analysis

After 24 h exposure, cells were re-seeded into 96-well plates at 1000 cells/well and 2000 cells/well. The viability was determined using Cell Counting Kit-8 (CCK-8, Dojindo Molecular Technologies, Inc., Kumamoto, Japan) at 0 (6 h after re-seeding), 1, 2, 3 or 4 days after seeding. CCK-8 reagent was added (10 µl per well) and incubated for an additional 4 h at 37°C. The optical density (OD) value of each well was measured using a microplate reader (TECAN, Männedorf, Switzerland) with a test wavelength of 450 nm. As a positive control, we treated cells for 1 h with 1 µM 4NQO to inhibit viability. The absorption was calculated as: Absorption = OD (experiment) - OD (blank). Each experiment was repeated three times and two dishes were included per exposure condition.

### Intracellular ROS Detection

The intracellular ROS level was measured by flow cytometry using 2′, 7′-dichlorofluorescin diacetate (DCFH-DA, Sigma). After exposure, cells were washed three times with pre-warmed serum-free medium and incubated in the presence of 5 µM DCFH-DA at 37°C for 20 min in dark. After washing 3 times to remove the extracellular DCFH-DA, cells were detached with 0.25% trypsin-EDTA, and the fluorescence intensities of the resuspended cells were measured by flow cytometry. For each sample, 10,000 cells were measured. As a positive control, we treated cells with 1 µM 4NQO for 1 h to induce cellular ROS. Each experiment was repeated seven times and two dishes were included per exposure condition.

### Statistical Analysis

All computations were performed with SPSS 16.0 for Windows. For the γH2AX formation, analysis was done using one-way analysis of variance followed by Dunnett’s two-tailed t-test. For the comet assay, cell cycle, cell proliferation, cell viability and ROS level, the two-tailed paired Student’s t-test was used to determine statistical differences between RF-exposed and sham-exposed groups, or between positive controls and sham-exposed groups. Data are presented as mean ± standard error of mean (SEM). A probability level of *p*<0.05 was considered statistically significant.

## Results

### RF-EMF Exposure Increases Cell Type-dependent γH2AX Foci Formation

**Figure 1:**
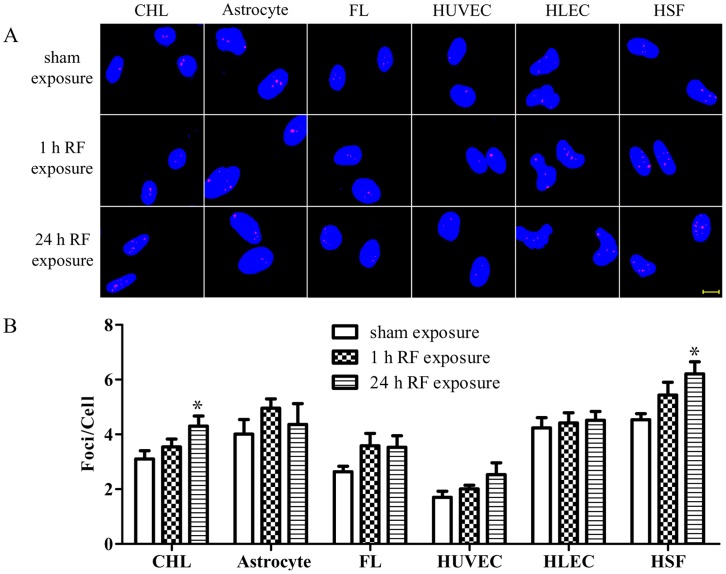
1800 MHz RF-EMF induces cell type-dependent DSBs as evaluated by the γH2AX foci formation assay. (A) Representative images of γH2AX immunofluorescent staining of CHL, astrocytes, FL, HUVEC, HLEC, and HSF exposed to radiation at 3.0 W/kg for either 1 h or 24 h. Red dots indicate γH2AX foci; nuclei are stained blue with DAPI. Scale bar, 10 µm. (B) Histograms showing the average numbers of γH2AX foci per cell by scoring ∼200 cells per sample. Values are mean ± SEM of at least 4 independent experiments. *p<0.05 compared with the sham-exposed sample.

Exposure to the RF-EMF for 1 h did not change the average number of foci per cell in each of the six cell types examined when compared to controls (Figure 1). After exposure to RF-EMF for 24 h, no significant changes in the average number of foci per cell were found between the exposure and sham-exposure groups in four of the six cell-types (FLs, HLECs, HUVECs, and newborn rat astrocytes) (Figure 1). However, the average number of foci per cell was significantly increased in CHLs after exposure to RF-EMF for 24 h when compared to sham-exposed cells (4.30±0.37 versus 3.10±0.30, *p = *0.022) (Figure 1). Similarly, the exposure significantly increased the average number of foci per cell in HSFs (6.21±0.44 versus 4.54±0.22, *p = *0.011) (Figure 1). These results indicate that RF-EMF induced γH2AX foci formation is cell-type dependent. As a positive control, 4NQO caused numerous, large, and bright foci in the nuclei of all six cell-types (Figure S2).

### RF-EMF-induced γH2AX Foci Formation does not Result in DNA Fragmentation

**Figure 2:**
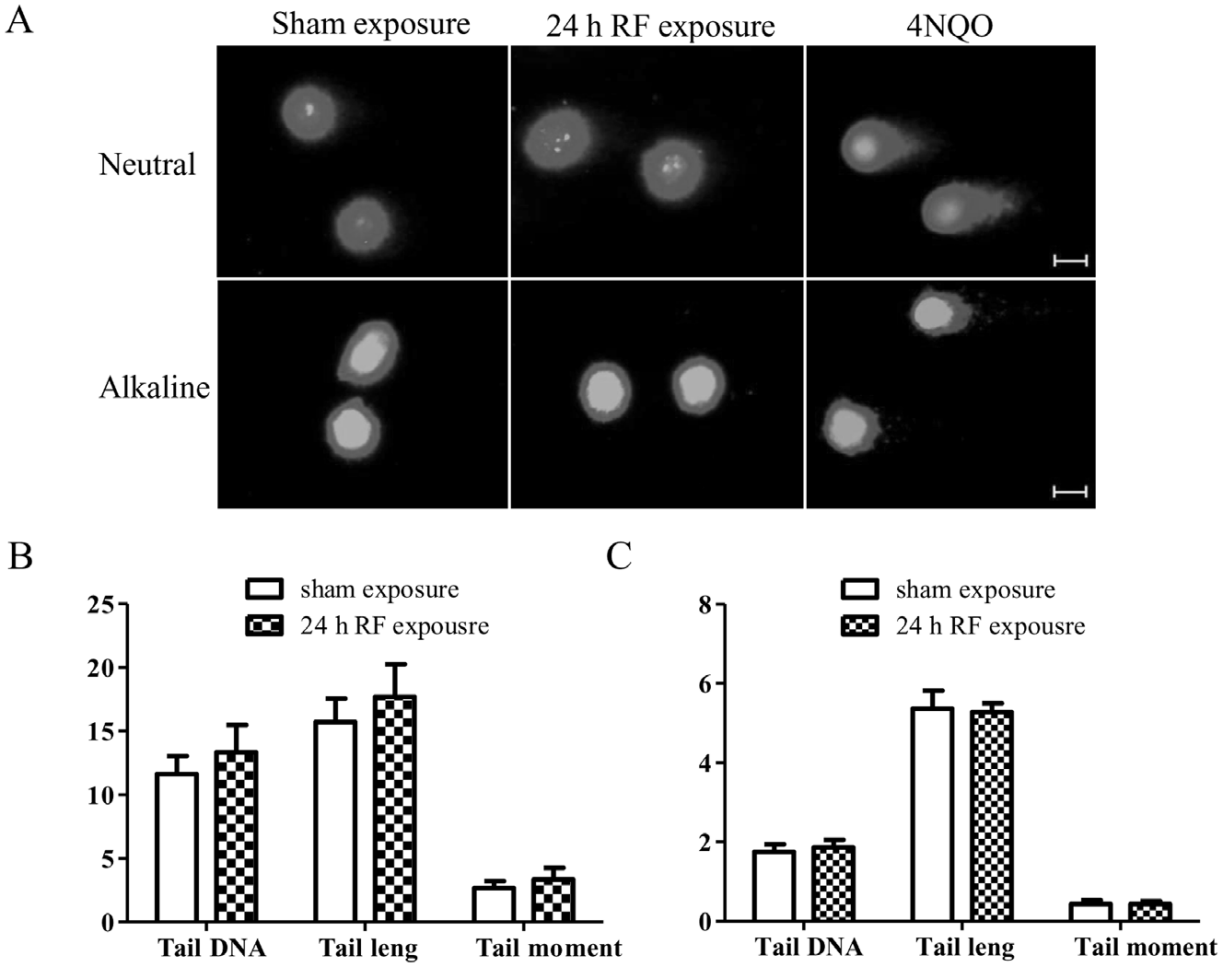
RF-EMF-induced γH2AX foci formation does not result in DNA fragmentation in HSF cells. (A) Representative neutral and alkaline comet images showing DNA fragmentation in HSF cells. Scale bars, 10 µm. (B, C) DNA fragmentation-induced tail DNA (%), tail length (µm) and tail moment (arbitrary units) in HSF cells after 24 h exposure in neutral (B) and alkaline (C) comet assay. Values represent mean ± SEM of 8 (neutral) or 12 (alkaline) independent experiments (Student’s t-test).

**Figure 3:**
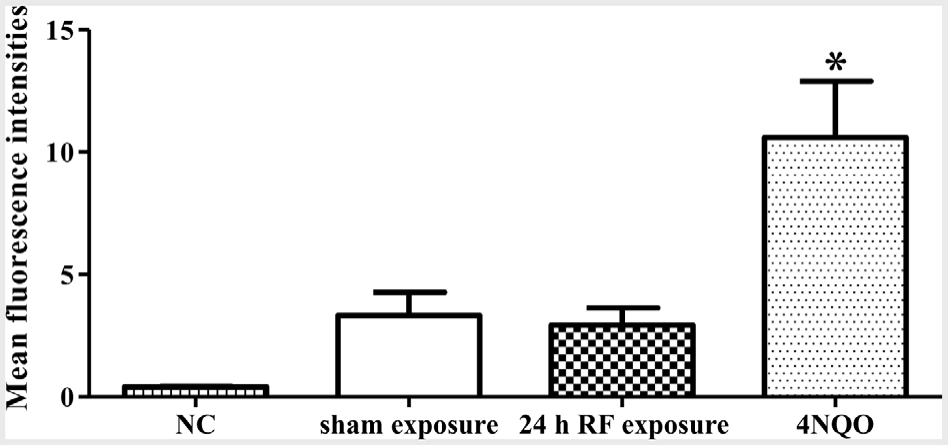
RF-EMF-induced γH2AX foci formation does not result in more DNA nicks in HSF cells. Histogram of DNA fragment levels in HSF cells. The background fluorescence value of the cells (NC) was determined without adding rTdT, and 1 µM 4NQO treatment for 1 h serves as positive control. Values represent mean ± SEM of 3 independent experiments. *p<0.05 compared with sham-exposed sample (Student’s t-test).

Since γH2AX foci formation indicates DSBs, we were interested in the biological consequences of this effect induced by RF-EMF. Therefore, HSFs were chosen as a model to determine whether RF-EMF exposure led to genetic instabilities, and/or aberrant cell cycle progression, proliferation and viability. First, we detected chromosomal DNA fragments using both comet assay and TUNEL assay in HSF cells. As expected, the 4NQO treatment induced dramatic DNA damages (Figure 2A & 3; Figure S3). After 24 h exposure to RF-EMF, however, no statistically significant differences were observed in the percentage of tail DNA, the tail length, and the tail moment between the sham and RF-EMF exposure groups as assessed by either alkaline (Figure 2B) or neutral comet assay (Figure 2C). TUNEL assay also revealed that there were no statistically significant differences in DNA damage (Figure 3; Figure S3).These results indicate that RF-EMF-increased γH2AX foci formation does not result in obvious chromosomal DNA fragmentation.

### RF-EMF-induced γH2AX Foci Formation does not Induce Sustainable Cell Cycle Arrest

**Figure 4:**
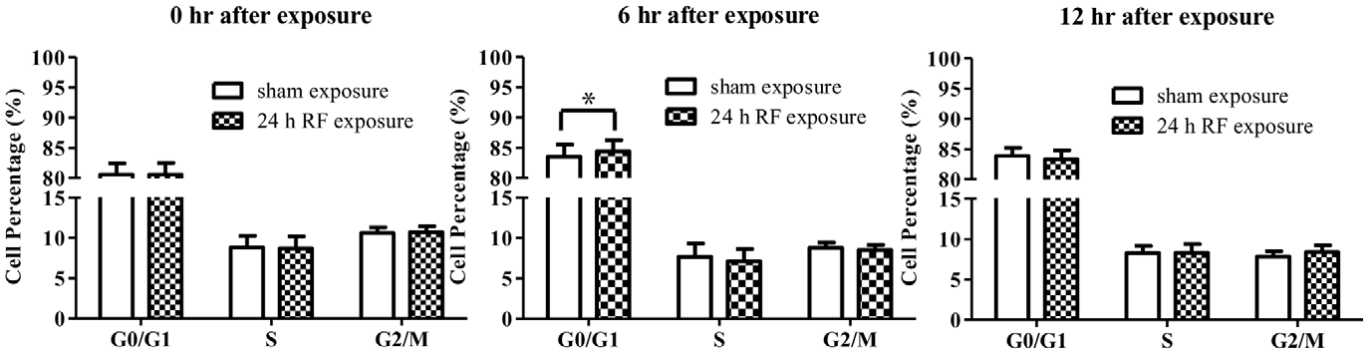
RF-EMF-induced γH2AX foci formation does not change cell cycle distribution in HSF cells. Histograms show the percentages of HSF cells in different phases of the cell cycle at 0 (left), 6 (middle), and 12 h (right) after 24 h exposure to 1800 MHz RF-EMF at 3.0 W/kg. Values represent mean ± SEM of 5 independent experiments. *p<0.05 compared with sham-exposed sample (Student’s t-test).

The accumulation of γH2AX foci is required to safeguard cell cycle checkpoints and arrest cell cycle progression to prevent cells from entering mitosis and also protect them from genomic instability [37]. Therefore, we next determined whether increased γH2AX foci formation in HSF cells affected cell cycle progression. Data revealed that the cell cycle distribution in HSFs was not affected immediately after the exposure (Figure 4). To evaluate the prolonged effects, the cells were incubated for another 6 or 12 h without RF-EMF exposure. The results showed that a slightly increased G0/G1 arrest occurred at 6 h after exposure as compared with the sham-exposed group (84.42±1.84% versus 83.52±2.03%, *p = *0.02), however, no significant changes of cell cycle progression were found at 12 h (Figure 4).

### RF-EMF-induced γH2AX Foci Formation does not Affect Cell Proliferation and Viability

**Figure 5:**
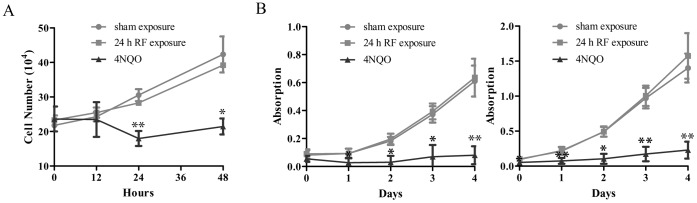
RF-EMF-induced γH2AX foci formation does not affect cell proliferation and viability in HSF cells. (A) HSF cell numbers at 0, 12, 24, and 48 h after 24 h exposure to 1800 MHz RF-EMF at 3.0 W/kg. (B) HSF cell viability at 0, 1, 2, 3, and 4 days after re-seeding at 1000 cells/well (B, left) and 2000 cells/well (B, right) immediately after 24 h exposure. 1 µM 4NQO treatment for 1 h serves as positive control. Values represent mean ± SEM of 3 independent experiments. *p<0.05 and **p<0.01 compared with sham-exposed sample (Student’s t-test).

To further investigate if the G0/G1 arrest affects cell growth, we measured HSF cell proliferation and viability at different time points after 24 h RF-EMF exposure. Proliferation was evaluated by cell counting. The results showed no significant difference in cell number between RF-EMF exposure and sham-exposure group just after the exposure (0 h, Figure 5A). Even the cells were incubated for another 12, 24, or 48 h without exposure, the proliferation rate was not changed (Figure 5A). To monitor cell viability, CCK-8 method was employed and no differences were found at different time points (1, 2, 3 or 4 days) after the exposure and at different re-seeding cell densities (1000 or 2000 cell/well) (Figure 5B; Figure S4). These data indicate that RF-EMF exposure does not affect cell proliferation and viability.

### RF-EMF Exposure does not Significantly Increase Cellular ROS Level

**Figure 6:**
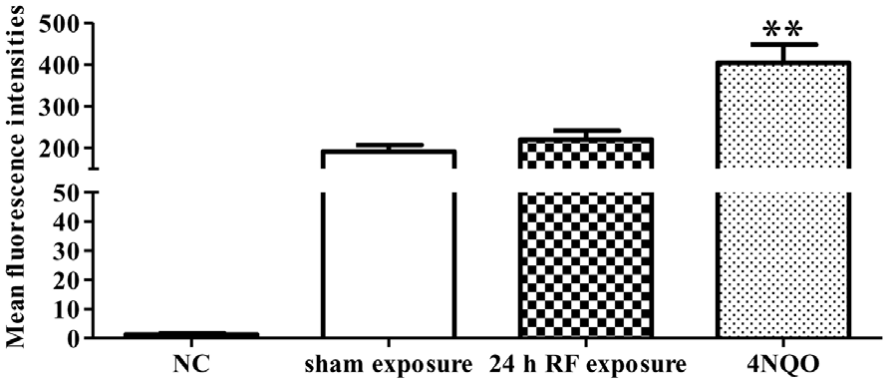
Effect of RE-EMF exposure on ROS production in HSF cells. Histogram of ROS levels in HSF cells. The intracellular ROS level of 24 h exposed cells was measured by flow cytometry using DCFH-DA. The background fluorescence value of the cells (NC) was determined without adding DCFH-DA, and 1 µM 4NQO treatment for 1 h serves as positive control. Values represent mean ± SEM of 7 independent experiments. **p<0.01 compared with sham-exposed sample (Student’s t-test).

It is generally accepted that the energy of EMF is not enough to damage DNA directly, thus indirect mechanisms, such as the free radical hypothesis, have been proposed to explain EMF-induced DNA damage [34], [38], [39]. We measured the intracellular ROS, and the results showed that RF-EMF exposure slightly but not significantly increased fluorescence intensity (220.14±20.74 in exposed versus 191.14±15.87 in sham, *p* = 0.216) (Figure 6). However, the 4NQO treatment significantly increased the ROS level in HSF cells.

## Discussion

It has been proposed that the inconsistency of data regarding the potential genotoxicity of RF-EMF exposure may come from the differences in exposure setups, experimental protocols, and biological systems. To address this concern, we investigated the effect of RF-EMF exposure on cellular DNA by exposing cells in an IT’IS-designed exposure system, visualizing DSBs with an identical γH2AX immunofluorescent staining protocol which was carried out by the same group of researchers, and comparing the effects in different cell types. There were six cell types tested in this study, i.e. CHL, newborn SD rat astrocytes, FL, HLEC, HSF, and HUVEC. CHL and HLEC were chosen to confirm the results of previous reports [19], [20]. The rat astrocytes and four human cell types represent different systems or organs, including the nervous system, reproductive system, visual system, defensive system, and endothelial system. We found that RF-EMF exposure significantly induced γH2AX foci formation in both CHL and HSF cells, but not in the other four types of cells, suggesting that the effects of RF-EMF on γH2AX foci formation is cell type-dependent. Further studies revealed that the cell type-dependent induction of DNA breaks did not result in significant biological changes in HSF cells.

The obtained results agree with the finding of Zhang et al. [20] where exposure to 1800 MHz RF-EMF at 3.0 W/kg for 24 h induced more γH2AX foci positive cells in CHL cells (percentage of γH2AX positive cells: 37.9±8.6% in exposed versus 28.0±8.4% in sham, *p*<0.05). Here we demonstrated that the mean number of foci per cell was also increased in 24 h exposed cells. Moreover, our data confirmed and extended the study of Yao et al. [19]. They found that exposure to 3.0 W/kg and 4.0 W/kg 1800 MHz RF-EMF for 2 h did not induced DSB in HLEC (percentage of γH2AX positive cells: 26.85±6.19% in 3.0 W/kg group, 27.97±6.19% in 4.0 W/kg group versus 25.29±5.44% in sham group, *p* >0.05). In the present study we revealed that exposure at 3.0 W/kg for 1 h or 24 h had no significant effect on DSB formation as well.

A number of studies have shown that different cells might respond differently to the same RF-EMF exposure [17], [18], [40]–[42]. However, the cell types applied in these studies were usually selected either randomly [42] or based on previous experience of the researchers [17], [18], [41]. Here we chose representative cells from five different systems. According to our knowledge, this is the first study to systematically investigate and compare the effect of RF-EMF on DNA damage in different cell types representing different systems or organs. Meanwhile, our finding illustrates that various cell model adopted by different research group may account for the inconsistent results.

Belyaev and his colleagues have employed γH2AX foci formation to investigate the effects of 905 MHz and 915 MHz GSM RF-EMF and universal global telecommunications system (UMTS) RF-EMF on chromatin structure and genome stability of lymphocytes, and found a stress response and DNA damage effect [17], [18]. Interestingly, they found that the exposures resulted in the reduced levels of 53BP1 and γH2AX foci in most of cells both from the so-called “hypersensitive” subjects and control individuals. The inconsistency between their studies and the present one could be explained by: 1) the difference of the frequency as we used 1800 MHz RF-EMF. Belyaev et al. did find a carrier frequency-dependent effect, and a statistically significant induction of γH2AX foci was observed in lymphocytes from a couple of donors in response to 905 MHz exposure [17], [18]; 2) the difference of RF-EMF exposure parameter. The Belyaev group adopted a transverse electromagnetic line cell (TEM-cell) which received signal from a test mobile phone, and continuously exposed the cells for 1 h. We used an IT’IS-designed RF-EMF cell exposure system and intermittently exposed the cells for 1 h or 24 h; 3) the difference of cell type. Therefore, it would be necessary to further systematically explore the differential effects of different exposure hardware, different exposure parameters (including frequency, duration of exposure, and exposure pattern etc.), and different cell types on DSB induction.

To further examine the biological consequences of RF-EMF-elevated γH2AX foci formation in HSF cells, we detected genetic stability, cell cycle progression and other cellular functions as the follow-up effects. The data showed that increased γH2AX foci formation did not resulted in irreversible DNA fragmentation and other cellular dysfunctions (i.e., sustainable cell cycle arrest, cell growth and viability change). Interestingly, we found that the positive control 4NQO at low concentration (0.05 µM) did induce γH2AX foci formation and inhibit cell proliferation; however, at extremely low concentration (0.01 µM) it induced γH2AX foci formation but without cell proliferation suppression (Figure S5). These data indicate that a slight increase of γH2AX foci formation does not affect cell behaviors even in a well-known chemical carcinogen. Although currently we do not know the mechanism of such phenomenon, it is possible that a slightly increased DNA damage (as indicated by γH2AX foci formation) induced by RF-EMF exposure might be repaired or compensated during cell progression. However, it is still possible that repeated insults of RF-EMF on the cells may result in unrepairable or mis-repaired DSB during a long period of exposure or under abnormal physiological conditions. Therefore, further studies using multiple rounds of RF-EMF exposure or under stress conditions of the γH2AX foci formation elevated HSF cells might be of interest.

Finally, we want to point out that the exposure setup used in the present study is different from the actual exposure of the real mobile telephony. Although the currently used exposure parameters mimick the basic pulse structure of the GSM signal at 1800 MHz and simulate the intermittency of real exposure by exposing the cells in an intermittent scheme, the parameters like intensity, frequency, modulation, etc, are still very different. Under a real exposure situation, these parameters keep constantly changing in unpredictable ways which may make a living organism more difficult to adapt. Further studies with real mobile phone signals would be warranted if a real-time dosimetry analysis could be achieved.

### Conclusion

The present study demonstrated that RF-EMF exposure induces γH2AX foci formation in a cell type-dependent manner. However, the DNA damage by the exposure might be reversible or compensated by DNA repair pathways or other cellular processes under the current experimental conditions. Based on past experience and the methodological challenges faced in EMF research, we propose that future experiments should be designed and carried out in a systematic and multi-center manner to guarantee the comparability, credibility, and reliability of the results, and if effects are detected by sensitive methods, studies should be extended to assess their biological consequences through an innovative way.

## Supporting Information

Figure S1 **Overview of the SAR distribution and thermal load.** (A) SAR distribution and (B) temperature distribution for the current study, where the average-SAR of the lowest medium layer of 50 µm thickness (monolayer) is 3.0 W/kg (non-uniformity <25%, k = 1; corresponding H^2^ in the waveguide 4.68 A^2^/m^2^) and the RF induced temperature increase stays below 0.08°C for steady state temperature. (C) Finite-difference time-domain (FDTD) simulation model [22].
(TIF)

Figure S2 **Effect of 4NQO on γH2AX foci formation in six cell types.** CHL, astrocytes, FL, HUVEC, HLEC, and HSF cells were sham-exposed or treated with 1 µM 4NQO for 1 h, and then subjected to γH2AX immunofluorescent staining. Representative images for each cell type showing γH2AX foci as red dots, and nuclei as blue which was stained with DAPI. Scale bar, 10 µm.
(TIF)

Figure S3 **Effect of RF-EMF exposure on DNA fragment formation in HSF cells.** TUNEL staining was assessed by flow cytometry. (A) The flow cytometer was gated to include single cells but to exclude any debris and clumps of cells according to the side and forward scatter patterns. (B) Representative histograms showed the background fluorescence value of the cells (NC) without adding rTdT, sham exposure group, 24 h RF exposure group, and positive control with 1 µM 4NQO treatment for 1 h.
(TIF)

Figure S4 **Effect of RF-EMF exposure on viability in HSF cells.** After 24 h exposure, the cell viability was examined at 0, 1, 2, 3, and 4 day(s) after re-seeding at 1000 cells/well (A) and 2000 cells/well (B). 1 µM 4NQO treatment for 1 h serves as positive control. Values are mean ± SEM of 3 independent experiments. *p<0.05 and **p<0.01 compared with sham-exposed sample (Student’s t-test). The cell viability rate was calculated as: Rate = Absorption (day of experiment)/Absorption (day 0).
(TIF)

Figure S5 **Effects of low dose 4NQO on DNA damage and proliferation of HSF cells.** HSF cells were exposed to 0.01, 0.02 or 0.05 µM 4NQO for 1 h, and then subjected to γH2AX immunofluorescent staining and cell counting. (A) Histograms showing the average numbers of γH2AX foci per cell by scoring ∼200 cells per sample. (B) Cell numbers at 0, 12, 24 and 48 h after 4NQO treatments of different doses in HSF cells. Values are mean ± SEM of at least 6 independent experiments. *p<0.05 and **p<0.01 compared with sham sample (Student’s t-test).
(TIF)
