# Supplementary figures and images for: Cell Type-Dependent Induction of DNA Damage by 1800 MHz Radiofrequency Electromagnetic Fields Does Not Result in Significant Cellular Dysfunctions
Source: PLoS One. 2013 Jan 23;8(1):e54906. doi: 10.1371/journal.pone.0054906 (PMC3552808; doi:10.1371/journal.pone.0054906)

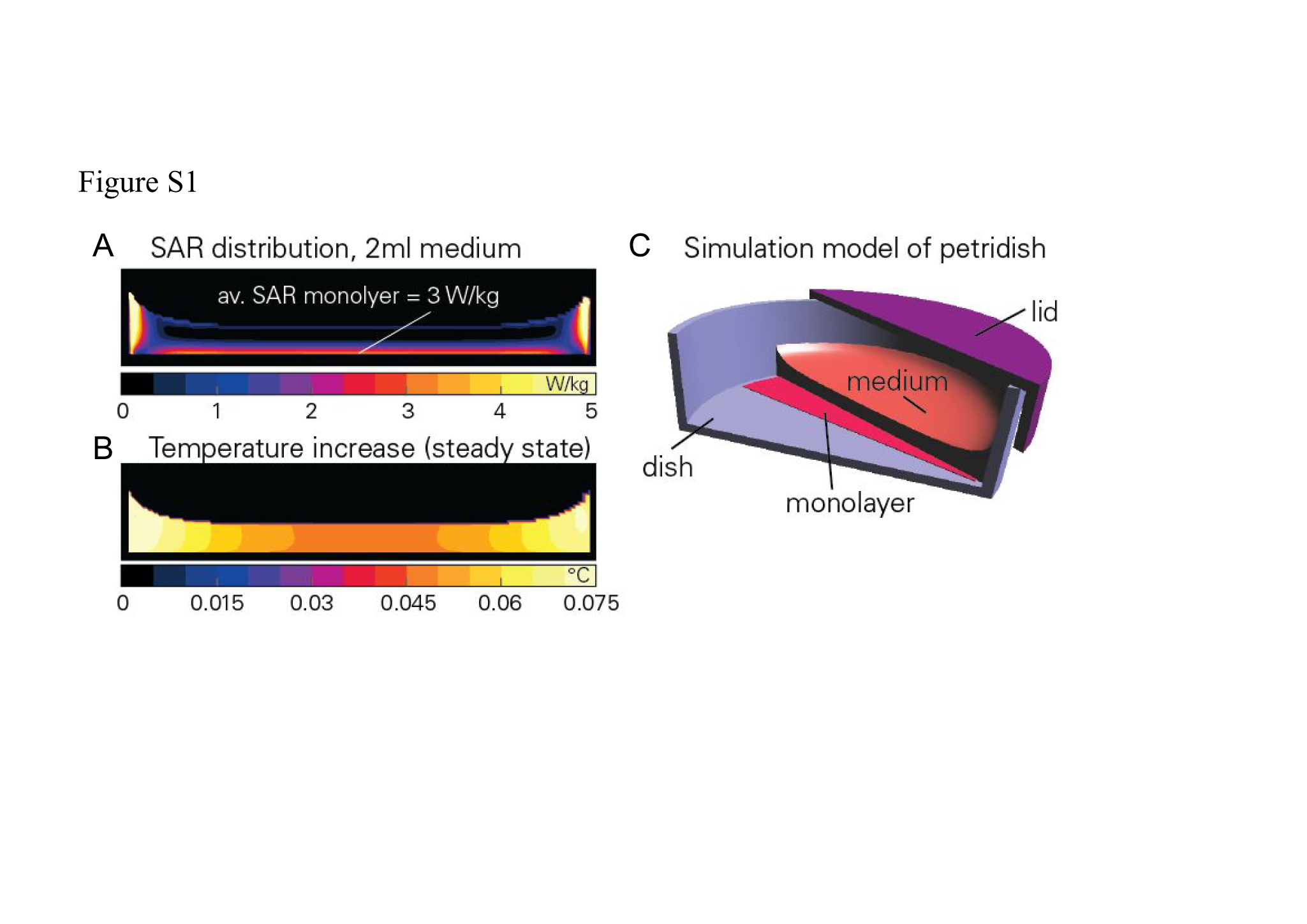

Supplement: Figure S1 — Overview of the SAR distribution and thermal load. (A) SAR distribution and (B) temperature distribution for the current study, where the average-SAR of the lowest medium layer of 50 µm thickness (monolayer) is 3.0 W/kg (non-uniformity <25%, k = 1; corresponding H2 in the waveguide 4.68 A2/m2) and the RF induced temperature increase stays below 0.08°C for steady state temperature. (C) Finite-difference time-domain (FDTD) simulation model [22]. (TIF) [file pone.0054906.s001.tif]

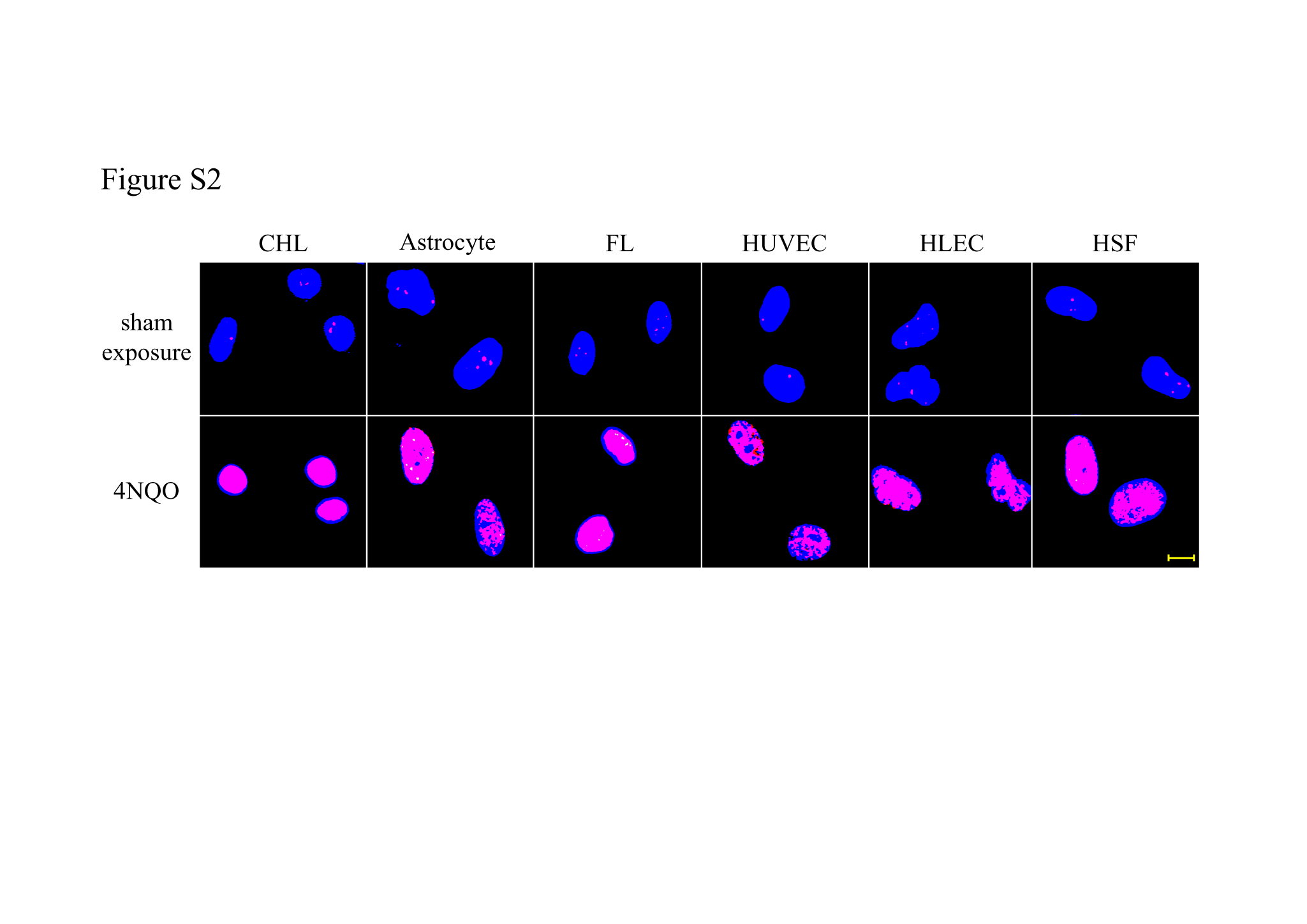

Supplement: Figure S2 — Effect of 4NQO on γH2AX foci formation in six cell types. CHL, astrocytes, FL, HUVEC, HLEC, and HSF cells were sham-exposed or treated with 1 µM 4NQO for 1 h, and then subjected to γH2AX immunofluorescent staining. Representative images for each cell type showing γH2AX foci as red dots, and nuclei as blue which was stained with DAPI. Scale bar, 10 µm. (TIF) [file pone.0054906.s002.tif]

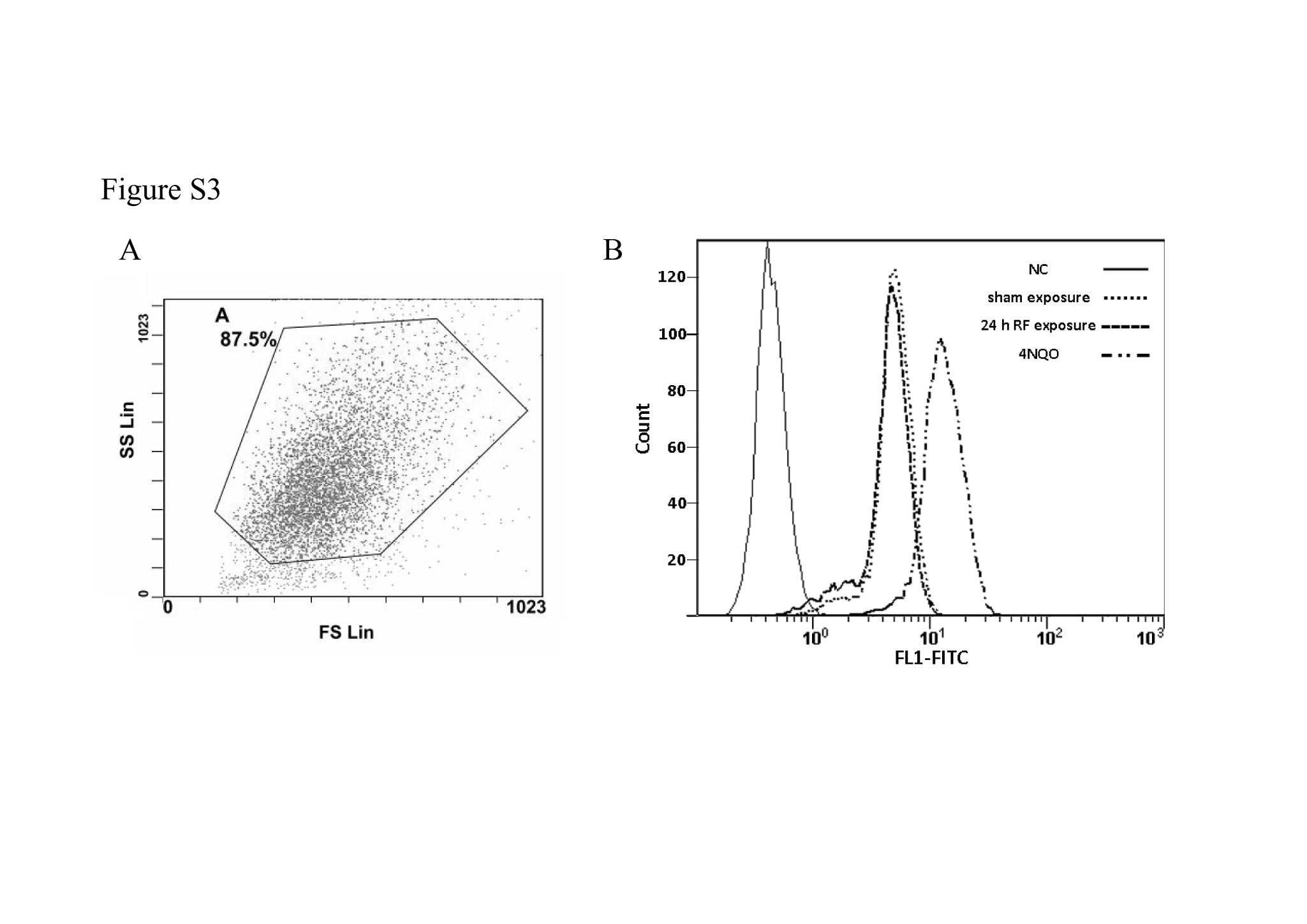

Supplement: Figure S3 — Effect of RF-EMF exposure on DNA fragment formation in HSF cells. TUNEL staining was assessed by flow cytometry. (A) The flow cytometer was gated to include single cells but to exclude any debris and clumps of cells according to the side and forward scatter patterns. (B) Representative histograms showed the background fluorescence value of the cells (NC) without adding rTdT, sham exposure group, 24 h RF exposure group, and positive control with 1 µM 4NQO treatment for 1 h. (TIF) [file pone.0054906.s003.tif]

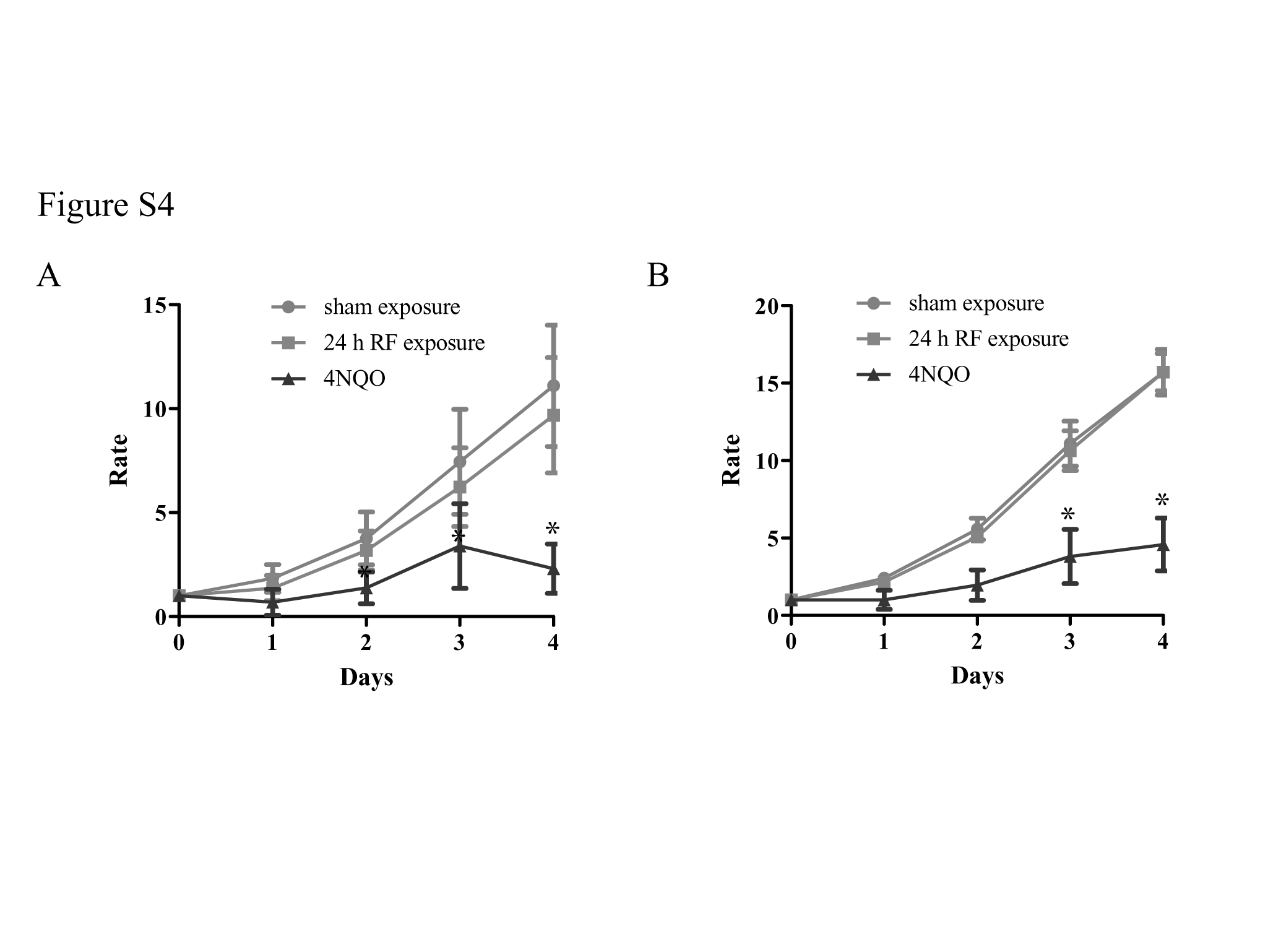

Supplement: Figure S4 — Effect of RF-EMF exposure on viability in HSF cells. After 24 h exposure, the cell viability was examined at 0, 1, 2, 3, and 4 day(s) after re-seeding at 1000 cells/well (A) and 2000 cells/well (B). 1 µM 4NQO treatment for 1 h serves as positive control. Values are mean ± SEM of 3 independent experiments. *p<0.05 and **p<0.01 compared with sham-exposed sample (Student’s t-test). The cell viability rate was calculated as: Rate = Absorption (day of experiment)/Absorption (day 0). (TIF) [file pone.0054906.s004.tif]

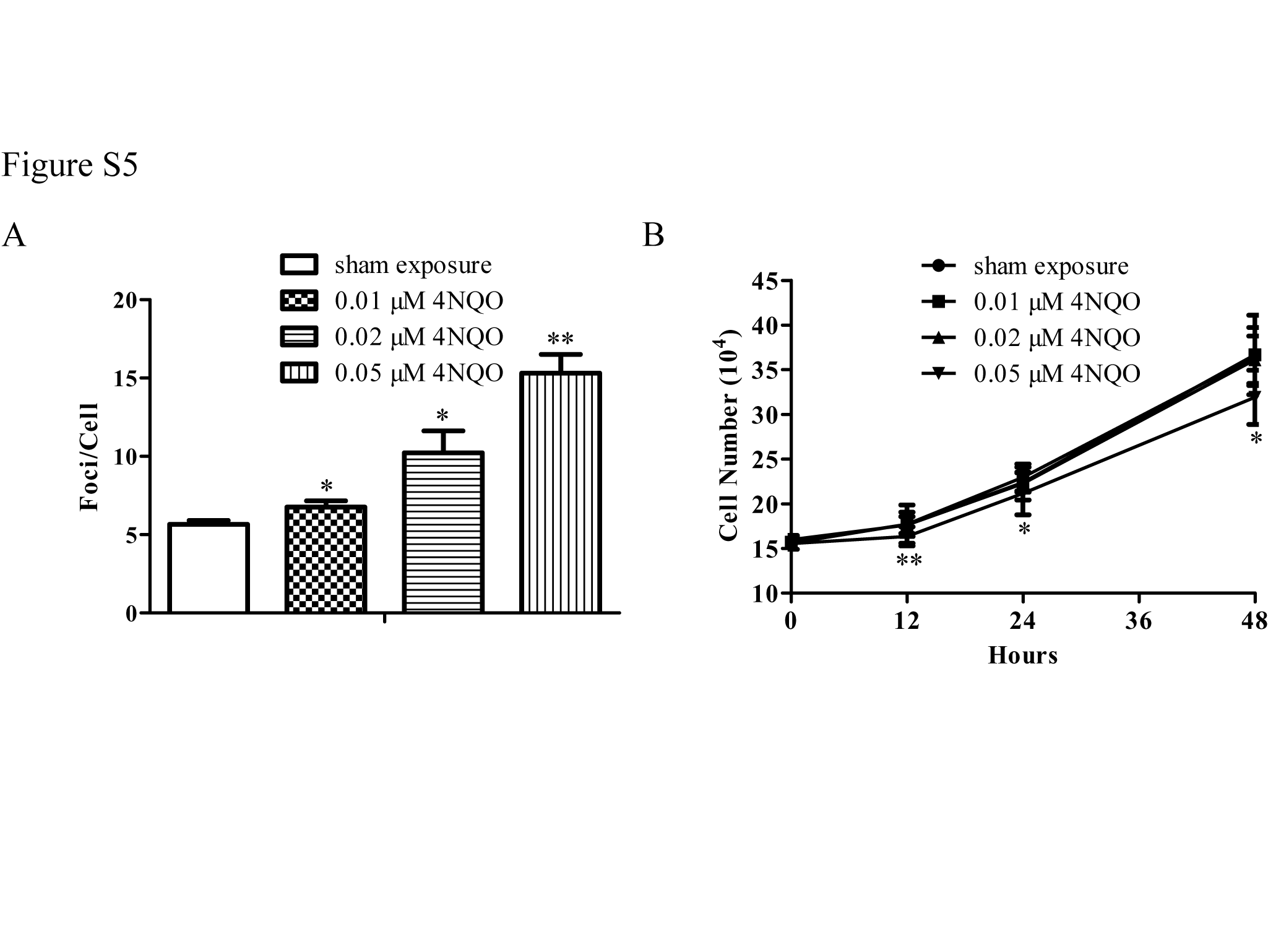

Supplement: Figure S5 — Effects of low dose 4NQO on DNA damage and proliferation of HSF cells. HSF cells were exposed to 0.01, 0.02 or 0.05 µM 4NQO for 1 h, and then subjected to γH2AX immunofluorescent staining and cell counting. (A) Histograms showing the average numbers of γH2AX foci per cell by scoring ∼200 cells per sample. (B) Cell numbers at 0, 12, 24 and 48 h after 4NQO treatments of different doses in HSF cells. Values are mean ± SEM of at least 6 independent experiments. *p<0.05 and **p<0.01 compared with sham sample (Student’s t-test). (TIF) [file pone.0054906.s005.tif]
